# IRDL Cloning: A One-Tube, Zero-Background, Easy-to-Use, Directional Cloning Method Improves Throughput in Recombinant DNA Preparation

**DOI:** 10.1371/journal.pone.0107907

**Published:** 2014-09-22

**Authors:** Jiancai Wang, Ronghua Xu, Aizhong Liu

**Affiliations:** 1 School of Life Sciences, University of Science and Technology of China, Hefei, People's Republic of China; 2 Key Laboratory of Tropical Plant Resources and Sustainable Use, Xishuangbanna Tropical Botanical Garden, Chinese Academy of Sciences, Kunming, People's Republic of China; 3 Kunming Institute of Botany, Chinese Academy of Sciences, Kunming, Kunming, People's Republic of China; University of Massachusetts Medical, United States of America

## Abstract

Rapid and efficient construction of expression vectors and subsequent transformation are basic recombinant methods for the investigation of gene functionality. Although novel cloning methods have recently been developed, many laboratories worldwide continue to use traditional restriction digestion-ligation methods to construct expression vectors owing to financial constraints and the unavailability of appropriate vectors. We describe an improved restriction digestion-ligation (IRDL) cloning method that combines the advantage of directional cloning from double digestion-ligation with that of a low background observed by using a positive selection marker gene *ccdB* to facilitate digestion and ligation in a single tube. The IRDL cloning overcomes the time-consuming and laborious limits of traditional methods, thereby providing an easy-to-use, low-cost, and one-step strategy for directional cloning of target DNA fragments into an expression vector. As a proof-of-concept example, we developed two yeast vectors to demonstrate the feasibility and the flexibility of the IRDL cloning method. This method would provide an effective and easy-to-use system for gene cloning and functional genomics studies.

## Introduction

Molecular cloning is an essential tool in modern molecular biology and biotechnology. With the rapid development of genomics in rapidly dissecting the functionality of specific genes, the requirement for constructing various vectors for gene transformation has become more acute than ever before. Therefore, developing a simple, efficient, universal, and cost-effective method for directional cloning of PCR products is particularly timely.

Traditionally, the cloning of an insert into an expression vector involves the use of Type II restriction enzymes to generate the appropriate DNA fragments encompassing the target sequence, followed by modification of DNA ends to generate blunt or sticky ends, and ligation of the DNA fragments to generate plasmids or other types of DNA vectors [1]. This procedure is extremely laborious and time consuming applied to a high-throughput format and is often limited by the relatively few restriction sites available [2]. Additionally, incomplete digestion of the vector reduces the efficiency of the screening process in identifying the single successfully cloned construct. Over the past two decades, a wide variety of novel cloning technologies, such as the Gateway system (Invitrogen), the Creator system and Infusion cloning (Clontech), LIC [3], TA cloning [4], MAGIC [5], SLIC [6], and Golden Gate cloning [7] have been developed, thereby improving cloning efficiency substantially. Gateway and Creator cloning systems are both site-specific recombination systems typically requiring a manual two-step process: i.e., inserting a target gene into a donor vector and subcloning the target gene from the donor vector into a recipient plasmid for subsequent expression. Like traditional cloning, the two-step cloning process is laborious and time consuming in a high-throughput setting, and the commercial recombinase is usually expensive. The MAGIC system, which uses *in vivo* recombination to transfer the gene from donor to recipient plasmid, decreased experimental costs substantially, but the experimental process remains laborious and time consuming. The LIC and SLIC methods use the exonuclease activity of T4 DNA polymerase to generate long cohesive ends between the vector and the target DNA fragments for subsequent annealing and *in vivo* repair. These methods require multiple experimental steps and long primers, which usually increase the cost and difficulty of PCR amplification. Infusion cloning enables easy construction of the expression vector *in vitro*, but also requires long primers. The TA cloning method, a one-step cloning process widely used for cloning PCR fragments into vectors, is rather limited in its construction of expression vectors since the insertion of fragments is non-directional, thereby increasing the labor in screening the correct clone. Golden Gate cloning is considerably faster and more accurate, with the potential of assembling multiple fragments in one pot and replacing the two steps of digestion and ligation with a single restriction-ligation step. Since this system is based on the action of the type II restriction enzyme BsaI, the backbone expression vectors require removal of internal BsaI sites [7].

Although the aforementioned cloning methods facilitate more efficient cloning, most laboratories worldwide continue to use traditional restriction digestion and ligation methods to construct expression vectors because of insufficient financial support and unavailability of appropriate vectors. Improvement of the restriction digestion-ligation methodology will increase the speed of the cloning process and save time, money, and labor. Here, we report an improved restriction digestion-ligation (IRDL) cloning method. This system combines the advantages of directional cloning of double digestion-ligation with that of a low background observed by using a positive selection marker gene to facilitate digestion and ligation in a single tube. Numerous cloning tests performed in our laboratory have shown that the IRDL cloning has a nearly 100% cloning efficiency. This system may be widely used for the insertion of PCR products into expression vectors in a one-step, single-tube digestion-ligation reaction.

## Materials and Methods

### Reagents and Genetic techniques

All basic DNA manipulation procedures were performed according to protocols described in Sambrook *et al*. [8]. The FastDigest restriction enzyme was purchased from Fermentas (Vilnius, Lithuania), the QuickCut restriction enzyme was purchased from TaKaRa (Kyoto, Japan), and the restriction enzyme with CutSmart buffer was purchased from New England BioLabs (Ipswich, MA, USA). T4 DNA ligase and ATP were purchased from Fermentas. High fidelity DNA Polymerase TransStart FastPfu (TransGen, Beijing, PR China) was used for DNA amplification. Plasmids were prepared using High-purity Plasmid DNA Mini-preparation Kit (Generay, Shanghai, PR China). The DNA concentration was measured with a NanoDrop Spectrophotometer ND-2000 (Thermo Scientific; Waltham, MA, USA). All plasmids used for IRDL cloning carry the gene *ccdB*, which is lethal to most host *Escherichia coli* strains; expression of the ccdB protein interferes with DNA gyrase and blocks the passage of DNA and RNA polymerases and leads to double-strand DNA breakage and cell death [9]. Plasmids containing the *ccdB* gene were propagated in the *E. coli* DB3.1 strain and plasmids without the *ccdB* gene were propagated in the *E. coli Trans*1-T1 strain (TransGen). All plasmids derived from PCR products were verified by sequencing. The strains and plasmids used in this study are listed in Table S1. Primers used in this study, synthesized by Generay, are listed in Table S2.

### Plasmids, IRDL cloning reaction, and transformation

The plasmid pWXY1.0 was constructed by replacement of the *URA3* autotrophic marker of p426-GAL with the *TRP1* marker by recombination in *S. cerevisiae* W303-1A and insertion of a 732-bp PCR-amplified *ccdB* cassette containing multiple cloning sites (MCS) from pCXSN by conventional cloning. The plasmid pWXY3.0 was constructed by replacement of the *TRP1* marker of pWXY1.0 with the *URA3* marker by recombination in yeast. To generate the constructs pWXY1.0-EGFP, pWXY1.0-JcDGAT1, pWXY1.0-JcPDAT1, and pWXY1.0-LacZ, *EGFP*, *JcDGAT1*, *JcPDAT1*, and *LacZ* flanked by the appropriate restriction sites were amplified from pXDG [9], p426-JcDGAT1 [10], pYES2.1-JcPDAT1 [11], and pYES2.1-LacZ (Invitrogen, Carlsbad, CA, USA) and subsequently inserted into pWXY1.0 by IRDL cloning. Details of these constructs were presented in Materials and Methods S1.

The standard IRDL cloning reaction mixture contained the following ingredients: 50 ng vector, the appropriate quantity of insert DNA in a 3∶1 molar ratio of insert to vector, 1 µL 10× FastDigest buffer, 0.5 µL 10 mM ATP, 0.25 µL FastDigest restriction enzyme 1, 0.25 µL FastDigest restriction enzyme 2, 0.25 µL T4 DNA Ligase, and distilled H_2_O up to a total volume of 10 µL. The IRDL cloning reaction mixture was incubated at 37°C or at 20°C for 30 min. Subsequently, 5 µL of the IRDL cloning reaction was transformed into 50 µL *Trans*1-T1 Phage resistant chemically competent cells (TransGen) according to the manufacturer's instructions. The transformation efficiency of *Trans*1-T1 Phage resistant chemically competent cells was 10^9^ cfu/µg of pUC19 DNA. The transformed cells were plated on LB plates containing 100 µg/mL ampicillin (Amp^+^).

To generate the *ScDGA1* fusion protein, the *ScDGA1* gene flanked by SpeI and AscI sites was amplified from *S. cerevisiae* genomic DNA with the primers DGA1-1 and DGA1-2 (Table S2). To generate the *EGFP* fusion protein, the *EGFP* gene flanked by AscI and XhoI sites was amplified from the plasmid pCambia35s-EGFP with the primers GFP-2 and GFP-7 (Table S2). After gel purification, approximately 50 ng of each PCR product was added to IRDL cloning digestion and ligation mixture contained 0.25 µL SpeI, 0.25 µL AscI, 0.25 µL XhoI, and 0.25 µL T4 DNA ligase. The reaction mixture was incubated at 37°C or 20°C for 30 min before *E. coli* transformation.

To generate the pWXY1.0-JcDGAT2 construct, the *JcDGAT2* gene was amplified from p426-JcDGAT2 [10] by two parallel PCRs with the primers pairs, P1–P2 and P3–P4 (Table S2), respectively. The PCR products were purified from agarose gels, and subsequently mixed together. Next, the PCR products were denatured by heating at 95°C for 10 min, followed by reannealing by cooling to room temperature. Concomitantly, a restriction digest was performed with 100 ng pWXY1.0, 1 µL 10× FastDigest buffer, 0.25 µL EcoRI, and 0.25 µL XhoI up to a 10 µL volume with distilled H_2_O, and incubated at 37°C for 5–10 min, followed by 80°C for 5 min. Next, 2 µL reannealed PCR products (100 ng/µL), 0.25 µL T4 DNA ligase, 0.25 µL 10 mM ATP, and 0.5 µL 10× FastDigest buffer were added to the digestion mixture up to a final volume of 15 µL with distilled H_2_O, and the mixture was then incubated at 25°C for 30 min before *E. coli* transformation.

### Yeast expression, TLC analysis, microscopy, and western blot

pWXY3.0-*ScDGA1*-*EGFP* was transformed into yeast quadruple mutant H1246α with the LiAc/SS Carrier DNA/PEG method [12]. Transformants were selected by growth on synthetic complete medium with the appropriate amino acid omitted and supplemented with 2% (w/v) glucose. Positive colonies were transferred into liquid SC media with the appropriate amino acid omitted and supplemented with 2% (w/v) glucose, followed by incubation at 28°C overnight. Overnight cultures were diluted up to an optical density (OD) of 0.4 in induction medium (SC-ura +2% galactose +1% raffinose), and induced for 24–36 h at 28°C. Cells were collected and lysed using glass beads and stored at −80°C for subsequent lipid analysis and protein extraction. Yeast lipid extraction, TLC analysis, microscopy and western blotting were performed as described previously [10], [13].

## Results

**Figure 1:**
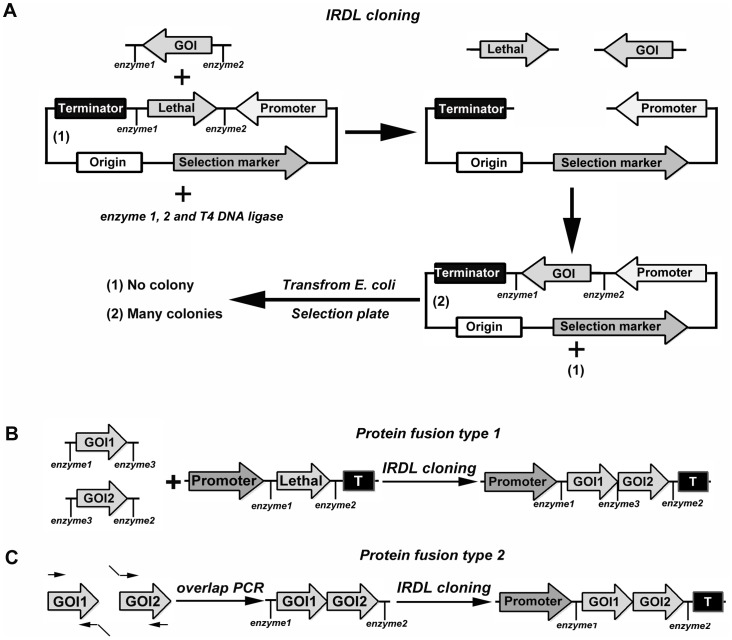
Schematic diagram of IRDL cloning. A, Construct expression of one insert by IRDL cloning. The purified PCR products of GOI and expression vector are mixed in a single tube together with restriction enzymes 1 and 2, as well as ligase. When the digestion and ligation of the mixture are in balance, two possible ligation products appear: (1) and (2). Only *E. coli* harbored the desired product (2) by its ability to survive on selection plates after transformation. Since the two different restriction enzymes used will generate two incompatible DNA ends, only the appropriate inserts can be ligated with the expression vector. B, Construction of fusion protein by IRDL cloning. The purified products of GOI1, GOI2, and expression vector are mixed in a single tube together with restriction enzymes 1, 2, and 3, as well as ligase. C, Construction of fusion protein by overlap extension PCR and IRDL cloning. GOI, gene fragment of interest. Lethal, lethal gene.

Since most projects in our laboratory require construction of a single expression vector, we often use a double-digestion and ligation method to generate expression vectors, but often encounter background problems because of incomplete digestion of the vector. To develop an easy-to-use, fast, efficient, directional, zero-background method, we inserted a lethal gene cassette flanked by two MCS (Fig. 1A). The expression vector also contained a selection marker, allowing for antibiotic selection, and an origin of replication in *E. coli*. The gene of interest was amplified by a high fidelity DNA polymerase with primers consisting of one available restriction site at each 5′ end. Since the two restriction enzymes used for cleavage of PCR products and expression vector were distinct, the overhangs after cleavage were incompatible. After gel-purification, the PCR products were mixed with the expression vector to perform a double-digestion and ligation procedure in a single tube. Once the digestion and ligation are in balance, two possible plasmids are present: the original expression vector and the desired recombinant plasmid (Fig. 1A). Because of the positive selection of the lethal gene, only *E. coli* harboring the desired construct could grow after transformation on selective media containing the appropriate antibiotics.

### One-step directional cloning of a single gene into an expression vector by the IRDL cloning

**Figure 2:**
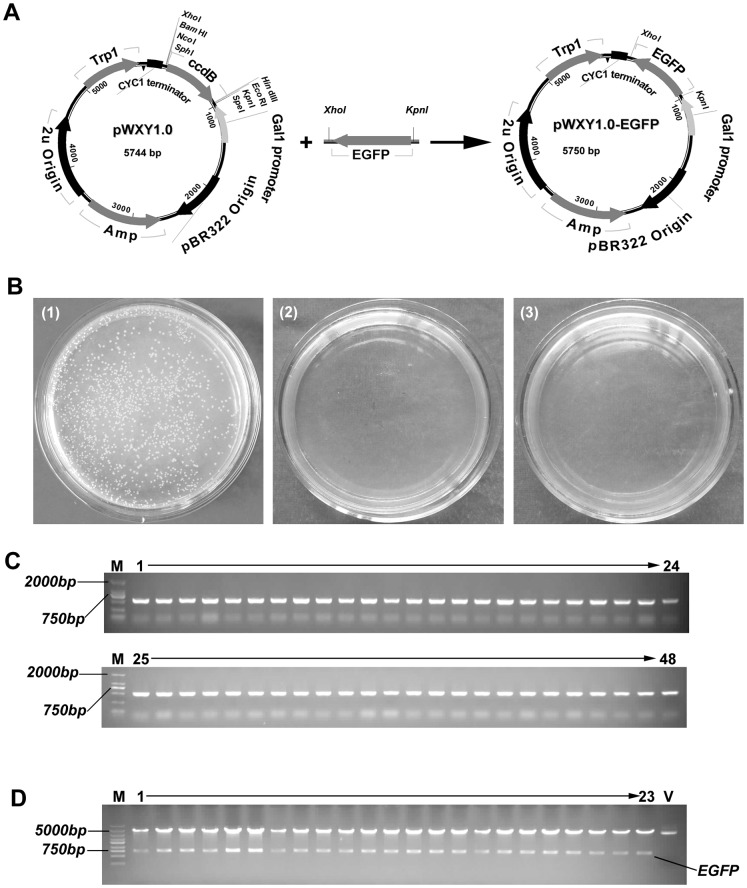
Subcloning of one insert into a yeast expression vector pWXY1.0 by using IRDL cloning method. A, Schematic representation of one-step directional cloning of EGFP into a yeast expression vector pWXY1.0. B, Test of the IRDL cloning system. (1): Cloning of EGFP into pWXY1.0 by standard IRDL cloning step (the purified PCR products of GFP and yeast expression vector pWXY1.0 are mixed in a single tube together with FastDigest buffer, restriction enzymes XhoI and KpnI, ATP and T4 DNA ligase, and incubated at 37°C for 30 min), followed by transformation into *E. coli Trans*1-T1 as described in materials and methods. (2): A control without T4 DNA ligase. (3): A control without restriction enzymes XhoI and KpnI. C, Colony PCR results from 48 recombinant colonies were run on 1% agarose gels. All of the colonies except the second clone tested contained the correct inserts. M, DNA ruler DL2501 from Generay. D, Plasmids DNA from 23 recombinant colonies and vector pWXY1.0 were digested with XhoI and KpnI and run on 1% agarose gels. DNA from all 23 recombinant colonies displayed the expected restriction pattern of pWXY1.0-EGFP. M, DNA ruler DL2502 (Generay). V, pWXY1.0.

To test the IRDL cloning system, we constructed a yeast expression vector pWXY1.0 (Fig. 2A). The pWXY1.0 contains a ccdB cassette for positive selection in commonly used *E. coli* strains (such as DH5α, TOP10), a *TRP1* autotrophic marker for selection in *S. cerevisiae* known as W303-1A, a pBR322 origin of replication for *E. coli*, a 2 *μ*origin of replication for *S. cerevisiae*, a glucose-depressed GAL1 promoter for inducing expression, and a CYC1 terminator. Two MCS are located at both ends of the *ccdB* cassette, each with four distinct restriction sites. To test the efficiency of the IRDL cloning system, we cloned the gene *EGFP* (0.7 kb) with the vector pWXY1.0. The gene *EGFP* was initially amplified by TransStart FastPfu DNA polymerase with primers GFP1 (containing the restriction KpnI site at 5′ end) and GFP2 (containing the restriction XhoI site at 5′ end) (Table S2). The PCR products of *EGFP* were gel-purified and were inserted into pWXY1.0 through the standard IRDL cloning reaction as described in materials and methods, and the reaction mixture were transformed to *E. coli* strain *Trans*1-T1. The results showed that the IRDL cloning system generated a large number of colonies, whereas both reaction mixtures (without T4 DNA ligase and restriction enzymes, respectively) as controls did not generate any colony on selective plate (Fig. 2B). Further, 48 colonies on LB plates were randomly sorted out for PCR analysis, resulting in the PCR products of all 48 colonies presented expected size bands (see Fig. 2C). Among these positive transformants, 23 of 48 colonies were subjected to restriction enzymatic analysis, leading to that the all colonies tested were true recombinants (Fig. 2D). The ten of these positive colonies was randomly selected and further confirmed by subsequent sequencing. These results showed that the IRDL cloning method was effective.

Apart from FastDigest restriction enzyme and digestion buffer (ingredients not disclosed by supplier), QuickCut restriction enzyme and digestion buffer (ingredients not disclosed by supplier) and restriction enzyme from New England BioLabs and CutSmart digestion buffer (50 mM potassium acetate, 20 mM Tris-acetate, 10 mM magnesium acetate, 100 µg/mL BSA, pH 7.9, 25°C) were also tested (Table 1). All the reaction mixtures tested were complemented with 0.5 mM ATP and 2.5 U T4 DNA ligase (Fermentas), incubated at 37°C for 5–30 min, and immediately transformed into competent *E. coli* cells, with transformants subsequently selected on LB Amp^+^ plates. For all of the reaction mixtures tested, more colonies appeared with longer incubation times. Restriction enzyme from different companies did display significant differences in colony number (ranging from 0.7–2×10^3^). Although the reaction mixture was incubated at room temperature (20°C), the results also displayed high cloning efficiency. Although cloning efficiency was enhanced by heat inactivation of the restriction enzyme after incubation prior to transformation and increased insert/vector molar ratios (data not shown), these actions were unnecessary for routine cloning. The results also demonstrated that as little as 5 min of the restriction-ligation step was sufficient to generate the desired construct for routine cloning into expression vector.

**Table 1:** Efficiency of cloning in pWXY1.0 under different conditions.

Buffer	Enzyme group	Insert	Colony number				Success rate
			5 min	10 min	20 min	30 min	
1×FastDigest	XhoI+KpnI	0.7 k/*EGFP*	264	382	587	1331	100%
1×QuickCut	XhoI+KpnI	0.7 k/*EGFP*	140	321	594	1005	100%
1×CutSmart	XhoI+KpnI	0.7 k/*EGFP*	410	752	1132	1857	100%
1×FastDigest	NcoI+SpeI	0.7 k/*EGFP*	209	360	635	985	100%
1×FastDigest	BamHI+EcoRI	0.7 k/*EGFP*	257	471	742	1437	100%
1×FastDigest	XhoI+KpnI	1.5 k/*JcDGAT1*	210	417	578	890	98%
1×FastDigest	XhoI+KpnI	2 k/*JcPDAT1*	173	312	598	932	96%
1×FastDigest	XhoI+KpnI	3 k/*LacZ*	109	230	420	731	92%
1×FastDigest	XhoI+KpnI	0.7 k/*EGFP* *	121	276	354	786	100%
1×FastDigest	XhoI+KpnI +AscI	2 k/*ScDGA1*+*EGFP*	75	180	360	720	100%

All reaction mixtures contained 1 mM ATP and 5 Weiss unit T4 DNA ligase (Fermentas) and incubated at 37°C. Experiments were performed using 50 ng vector, and the corresponding amount of insert DNA at a 3∶1 molar ratio of insert:vector. None of the reaction mixtures here was inactivated with heat prior to *E. coli* transformation.

* indicates the reaction mixture was incubated at room temperature 20°C. Cloning efficiencies were given as colonies number on LB plates containing 100 µg/mL ampicillin. Cloning success rate were evulated by colonies PCR test of 50 radom seleted colonies. Specific primers of different inserts used as forward primer, and CYC1 primer in CYC1 terminator used as a reverse primer.

To examine the cloning efficiency of IRDL cloning with different insert fragments in length and incubation time, aside from *EGFP*, the genes *JcDGAT1* (1.5 kb), *JcPDAT1 (*2 kb), and *LacZ* (3 kb) were further tested. Similarly, these fragments were amplified by high fidelity DNA polymerase with specific primers containing the appropriate restriction sites (Table S2), and then the PCR products were purified and cloned into pWXY1.0 following the standard IRDL cloning steps with FastDigest restriction enzyme and digestion buffer. As shown in Table 1, the generated colony number increased with the elongation of incubation times in all cases, whereas with the increasing of the insert fragments in length the colony number decreased. When the insert fragment was 3 kb (*LacZ*) the colony number varied from 109 to 731. When the insert of combined *EGFP* and *ScDGA1* was used the IRDL cloning produced 75 colonies after 5 min incubated. These colonies were sufficient to satisfy the general requirement in most laboratories. Further, the PCR inspection with gene specific primers confirmed the positive clones, resulting in a high cloning efficiency from 92%, 96% to 98% for *LacZ*, *JcPDAT1 and JcDGAT1*, respectively.

### Generation of fusion protein by IRDL cloning

**Figure 3:**
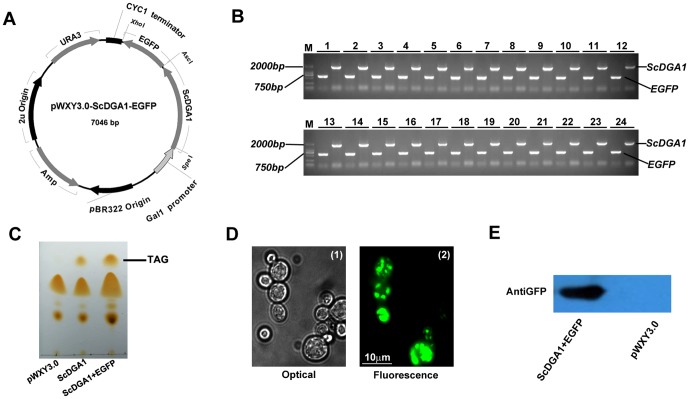
Fusion of two inserts, ScDGA1 and EGFP, into a yeast expression vector pWXY3.0 using IRDL cloning. A, The plasmid pWXY3.0-ScDGA1-EGFP generated by using IRDL cloning method. B, Colony PCR results from 24 recombinant colonies were run on 1% agarose gels. All of the colonies tested contained the corrected inserts. M, DNA ruler DL2501 (Generay). C, TLC separation of neutral lipids from different yeast strains. The transgenic H1246α with the *ScDGA1* and *ScDGA1-EGFP* restore triacylglycerol (TAG) synthesis compared with negative control (H1246α with pWXY3.0). D, Fluorescence microscopy of ScDGA1-EGFP fusion protein expression in transgenic yeast cells. E, Detection of ScDGA1-EGFP fusion protein by western blotting.

Expression of a fusion protein is a widely used methodology for rapid and effective characterization of gene product function. To test the ability of the IRDL cloning system in cloning two fragments concomitantly, we generated a *ScDGA1* and *EGFP* fusion plasmid (Fig. 3A) for overexpression in yeast. Two methods exist to construct a fusion protein (Fig. 1B and 1C). The first requires the use of restriction enzymes and ligases, and the second employs overlap extension PCR. As the cloning of overlapping PCR products was expected to be similar to one-insert cloning, we tested the efficiency of assembly of two inserts, *ScDGA1* and *EGFP* in pWXY1.0, by digestion with three restriction enzymes. The restriction-ligation mixtures were prepared as previously described as one-insert cloning by using FastDigest restriction enzymes. After gel-purification, the two PCR products *ScDGA1* (containing a 5′ SpeI site and a 3′ AscI site) and *EGFP* (containing a 5′ AscI site and a 3′ XhoI site) were added to the mixture and incubated at 37°C for 5–30 min. The transformation and screening steps were performed as described above. Although the number of colonies was significantly lower than that during the cloning of a single insert, the 75 colonies obtained after a 5-min reaction period were sufficient to satisfy the requirement of fusion construction. PCR analysis of 24 randomly selected colonies from LB Amp^+^ plates indicated that all colonies tested contained the expected construct (Fig. 3B), with 10 of those colonies confirmed to contain the insert by subsequent sequencing. One colony was selected for plasmid purification and introduced into the *S. cerevisiae* mutant H1246α for *GAL1*-inducing expression. As a *dga1Δlro1Δare1Δare2Δ* quadruple mutant (QM) strain, H1246α lacks all four enzymes for neutral lipid synthesis, and triacylglycerol (TAG) was not observed in this strain [14], [15]. Therefore, H1246α is extensively used in characterization of the gene encoding of diacylglycerol acyltransferase (DGAT) enzyme [10], [15]. As shown in Fig 3C, overexpression of ScDGA1-EGFP fusion protein restored TAG synthesis similar to overexpression of the *ScDGA1* alone, indicating that the fusion protein has comparable functionality to DGAT enzyme. Expression of *ScDGA1-EGFP* was also confirmed by GFP fluorescence (Fig. 3D) and western blotting (Fig. 3E).

### Creating expression clones free of restriction site limitations

**Figure 4:**
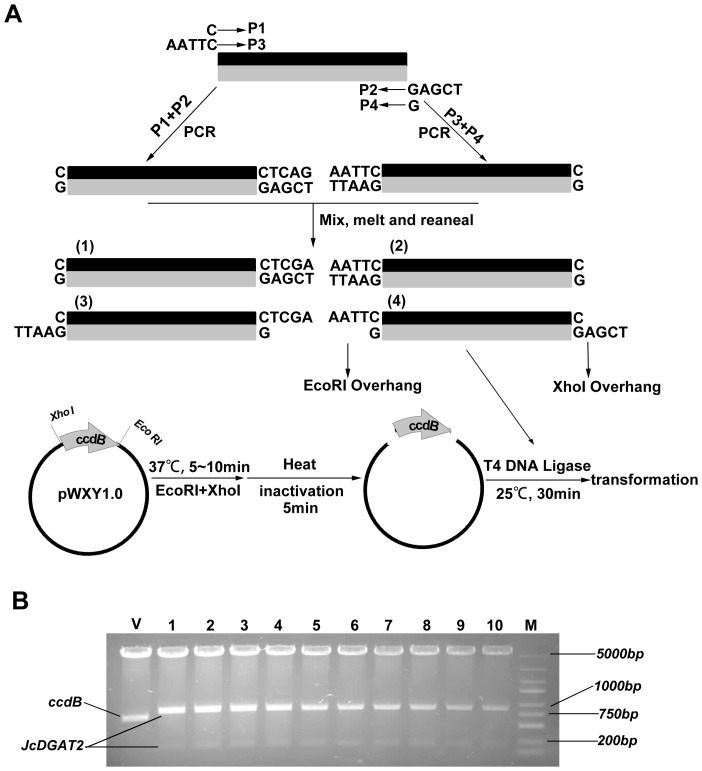
Schematic diagram of cloning the gene of interest containing internal restriction sites into expression vector. A, generation of sticky-end fragments and cloning into pWXY1.0 by IRDL cloning. The *JcDGAT2* was amplified by two pairs of primers, P1–P2, and P3–P4, which were appended with short sequence tails: C, TCGAG, AATTC, G, respectively. After gel-purification, the two PCR products were mixed together, denatured, and reannealed, resulting in 25% of the DNA fragments with Eco*RI* and XhoI overhangs. Concomitantly, the vector was double-digested with EcoRI and XhoI for 5–10 min at 37°C. After heat inactivation of the restriction enzyme at 95°C for 5 min, the vector was mixed with the reannealed JcDGAT2 containing EcoRI and XhoI overhangs, T4 DNA ligase and ATP were added and incubated at room temperature for 20 min, and finally transformed into *E. coli*. B, Restriction digestion (EcoRI and XhoI) of minipreps of pWXY1.0 (V) and pWXY1.0-JcDGAT2 (lane 1 to lane 10). The restriction pattern of pWXY1.0-JcDGAT2 generated by EcoRI and XhoI digestion was as predicted: 861 bp and 200 bp, respectively. M, DNA ruler DL2502 (Generay).

One limitation of the IRDL cloning method may stem from the occasional presence of one or more internal restriction site(s) in the gene of interest, thereby precluding the use of this strategy. A general solution to this challenge is the use of two pairs of primers to generate PCR products with sticky ends through enzyme-free cloning [16], [17], which could be complementary with a double-digested quick-clone plasmid. Such cloning requires performance of the double digestion first, followed by inactivation of the double restriction enzyme by heat inactivation, addition of the PCR products with sticky ends, and finally performance of ligation. To test this strategy, we cloned a *Jatropha curcas DGAT2* gene (GenBank accession number: HQ827795), which contains a single internal EcoRI site, into plasmid pWXY1.0. To generate PCR products with EcoRI and XhoI overhangs, two parallel PCR amplifications of *JcDGAT2* were performed following the strategy illustrated in Figure 4A. Primers P1, P2, P3, and P4 were appended with a tail containing the following sequences: C, TCGAG, AATTC, and G, respectively. The two PCR products were gel-purified, mixed in equal volume, and denatured by heating to 95°C, followed by cooling to room temperature, thereby causing the double-stranded DNA molecules to denature and reanneal. This process results in 25% of the DNA molecules containing EcoRI and XhoI overhangs (Fig. 4A). To avoid EcoRI cleavage of the *JcDGAT2*, the plasmid pWXY1.0 was double-digested with EcoRI and XhoI, followed by 85°C heating for 5 min to inactivate the restriction enzyme. The reannealed *JcDGAT2* PCR products, T4 DNA ligase, and ATP were added to the restriction mixture and incubated at room temperature for 20 min, followed by *E. coli* transformation. Colony PCR analysis and restriction analysis of 10 randomly selected colonies indicated that all colonies contained the expected construct (Fig. 4B), confirmed by subsequent sequencing.

## Discussion

Conventional cloning methods based on restriction digestion and ligation have played a critical role in the construction of recombinant DNA molecules. However, because of its laborious and time-consuming nature, as well as limitations in restriction sites in the MCS, a wide variety of novel cloning technologies have been developed as alternate methodologies to restriction digestion and ligation. Among these technologies, the Gateway system has been the most popular choice for vector construction in the research community for its convenience for DNA transfer into multiple expression vectors [18]. Additionally, many Gateway-compatible entry clone collections and expression vectors have been generated for a multitude of species [19], [20], [21], [22], [23]. For thousands of individual research groups worldwide, the Gateway system is not practical, since most projects only require the construction of a single expression vector. Moreover, the Gateway system has limited utility for investigators working on non-model organisms, since no entry clones and expression vectors are available. To overcome these limitations in both traditional methods and the Gateway system, we developed a substantially improved technique over the traditional restriction digestion-ligation method, referred to as IRDL cloning. A lethal marker gene (*ccdB*) flanked by multiple distinct restriction sites was introduced into the MCS so that PCR inserts and their backbone vectors could be subjected to restriction digestion and ligation in a single tube. With the positive selection of the *ccdB* gene and the incompatible ends of the vectors, any undigested backbone vector and self-ligation transformants were eliminated, and only correct and directional cloning results were able to survive on the selection plates. Here, we developed two yeast expression vectors to validate our system. The results showed that a gene or DNA fragment of interest can be easily cloned into an expression vector by a one-step procedure, confirming that our system is an easy-to-use, efficient, rapid, zero-background, directional cloning method. With minor modifications (e.g., combined with infusion PCR or an enzyme-free cloning method), our system can also be used to clone DNA fragments with occasional internal restriction site.

Compared to Gateway and other systems, our system possesses several advantages: (a) our system required no entry clones, and only one-step incubation, followed by standard transformation was required. It frees investigators from laborious cloning processes. By contrast, conventional restriction digestion-ligation, Gateway, and LIC all require multiple cloning procedures. Although the TA cloning method could rapidly clone PCR products, preparation of the TA vector involves multiple steps and the final TA-generated construct typically possesses no directionality, resulting in difficulties in selection [24]. TOPO cloning methods also perform rapid cloning, but lack directionality. Although available from commercial sources (Invitrogen and TransGen), no reports are currently published on the development of high-quality expression vectors based on TOPO cloning in individual laboratories, probable related to technology difficulties. (b) Our system does not require long primers for PCR amplification. By contrast, the Gateway, Infusion, and LIC all require long primers, which commonly increase cloning difficulties and cause errors in PCR amplification [25], in addition to increased costs as the number of constructs multiply. (c) Our system does not depend on expensive recombinases, thereby saving on cost. Although a variety of tested Gateway expression vectors are available from Invitrogen and from the research community at large, many small laboratories are unwilling to use this technology, since the Gateway recombinase is expensive. Even laboratories that can afford the cost of this system may elect to scale down their reactions to a 5-µL volume. The cost of recombinase is also often prohibitive in the Infusion kit and the like, such as CloneEZ (Genescript, Nanjing, PR China), Cold Fusion Cloning (SBI, Mountain View, CA, USA), Fast Seamless Cloning (DoGene, Shanghai, PR China), and GENEART Seamless cloning (Invitrogen), pEASY-Uni Seamless Cloning (TransGen, Beijing, PR China), especially when used for a large-scale protein expression and purification studies. Zhang et al. [1] used crude bacterial extract containing recombinase to perform seamless cloning by *in vitro* recombination, but the preparation of crude recombinase extract is a multistep process requiring some skill. By contrast, we used conventional restriction enzymes to perform IRDL cloning. Considering that the cloning process requires only small volume of enzymes, the cost is affordable for the standard academic laboratory. (d) Compared to the more complex development of plasmids based on Gateway, LIC, and TA systems, the plasmids used in the IRDL cloning strategy for different research purposes are easy to develop. The insertion of a lethal gene cassette flanking multiple single restriction sites into the backbone plasmid may complete the construct. (e) The expression plasmid used in IRDL cloning does not require linearization or any modification. By contrast, the plasmid used for Infusion cloning requires linearization and purification [2] and the vector used in the TA system requires prior preparation. Unfortunately, the 3′-dT overhang of the TA system is unstable and usually absent during storage (see PCR cloning protocol, [26]). Since the plasmids are unimpaired before digestion, the expression vectors used in IRDL cloning are very stable, even during long-term storage. (f) The Flexi Cloning system (Promega) used lethal gene marker *barnase*, two rare restriction enzyme SgfI and PmeI, and antibiotic resistance change to facilitate the shuttling of gene of interest between the different Flexi Vectors [27]. Like the traditional restriction digestion and ligation methods, cloning of PCR products into the entry Flexi Vectors also involved multiple steps (See Technical mannual of Flexi Vector System, http://www.promega.com.cn/techserv/tbs/TM001-310/tm254.pdf), such as restriction digest of PCR products and acceptor Flexi vector, cleanup of the PCR products, ligation of PCR product and acceptor Flexi vector. However, our IRDL cloning method simplified these experimental steps into one step and one tube.

The *ccdB* gene encodes a lethal protein, which impairs topoisomerase II (DNA gyrase) activity, resulting in irreparable DNA damage and death of the host *E. coli*. Since cells containing the gyrA462 mutation and cells containing the F′ episome carried a *ccdA* gene encoding for an antidote to the *ccdB*-expressing toxic protein, they are not sensitive to its *ccdB*-killing activity [28]. The *ccdB* and *ccdA* of F′ episome expressed a toxin-antitoxin construct that has been extensively used in the construction of various plasmids with positive selection [9], [24], [25], [29], [30], including commercial Gateway plasmids. Besides the *ccdB* lethal marker, other lethal genes can be used as positive selection marker, e.g., the cell lysis-related genes from phages, Mu, X174, and Q [28]; the plasmid-encoding toxin, e.g., RelE, PemK/Kid, Doc, ShoB, Zor, and MazF [31], [32], [33], [34]; and other lethal genes, e.g., *Hlg1* [35], *RCSB* [28] and *barnase* [27]. Although the precise mechanisms for their action are not well understood, these lethal genes should theoretically function properly in the IRDL cloning system. Recently, Mok and Li [36] developed a similar cloning method, referred to as the detox system, but they did not combine the digestion and ligation protocols into a single step. Therefore, the resulting procedure is 20 times as long as the IRDL cloning procedure (30 min digestion and 70 min ligation for detox cloning versus 5 min digestion and ligation for IRDL cloning). Furthermore, we presented a remedy for cloning a gene of interest containing an internal restriction site.

The IRDL cloning system is a powerful tool that is unrestricted to cloning a functional gene. Any genetic elements with decoding sequence information—including promoters, terminators, attenuators, enhancers, and origins of replication—can also be cloned using this method. Moreover, the IRDL cloning strategy is easy-to-use in constructing plasmids for different research purposes, such as constitutive expression, transient expression, gene silencing, protein tagging, promoter activity, and protein subcellular localization in bacteria, fungi, plants, insects, and mammals. The cloned fragments can also be cleaved and transferred to another vector based on the change of antibiotic marker. However, the IRDL cloning method has a drawback when restriction sites flanking the lethal marker occurred in the target gene of interest, which would preclude its application. To overcome this problem we proposed eight different restriction sites on both sides of the lethal marker gene, which could provide with 16 possible combinations for restriction digestion, might be usually sufficient to clone a given gene. In addition, IRDL cloning is developed based on the double restriction digestion for cloning a given gene. In particular, when one constructs an expression vector for analyzing the function of a given gene, IRDL cloning is quite helpful.

Recently, several seamless cloning methods such as QC (Quick and Clean) cloning, One-step SLIC (Sequence- and Ligation-Independent Cloning) and FastCloning have been developed [37], [38], [39]. These methods substantially improve traditional cloning techniques, greatly facilitate cloning work because their easy-to-use, fast and simple features. However, the three methods require long primers for PCR amplification compared to IRDL cloning method, which may increase the cost and cause errors in PCR amplification as described above. In addition, both QC cloning and one-step SLIC need linearize vectors before cloning. The FastCloning is often confined when constructing a large vector (such as a plant expression vector, larger than 12 kb often) in length because this method requires to amplify the vector. In contrast, QC cloning seems be more suitable for cloning specific PCR products from unspecific mixes; one-step SLIC may be more helpful for assembly multiple fragments; FastCloning may be more usable for constructing expression vector, chimeras and producing multiple mutations. IRDL cloning can be more helpful for constructing a given expression vector. For cloning an uncertain PCR fragment using some known sequences, such as PCR products from TAIL PCR, adaptor PCR and RACE PCR, those methods independent of restriction enzyme such as Gateway, Infusion, TA, and LIC would be more helpful.

In summary, the low cost, convenience, and high efficiency of the IRDL cloning system will have a potential for wide application and facilitate plasmid construction and rapid speed for functional genomics studies. Even laboratories with modest budgets can use this system to perform high-throughput cloning or preparation of expression constructs for functional genomics research. This method is a useful alternate methodology to existing cloning methods based on recombination.

## Supporting Information

Table S1 **Strains and plasmids used in this study.**
(PDF)

Table S2 **Primers in the study.**
(PDF)

Materials and Methods S1 (PDF)
