# Supplementary material for: IRDL Cloning: A One-Tube, Zero-Background, Easy-to-Use, Directional Cloning Method Improves Throughput in Recombinant DNA Preparation
Source: PLoS One. 2014 Sep 22;9(9):e107907. doi: 10.1371/journal.pone.0107907 (PMC4171505; doi:10.1371/journal.pone.0107907)
Supplement: Table S1 — Strains and plasmids used in this study. (PDF) [file pone.0107907.s001.pdf]

Table S1

Strains and plasmids used in this study

Strains or plasmids	Source or referenece
**Strains**	
*E. coli Trans*1-T1	Transgen, Beijing, China
E. coli DB3.1	(25)
S. cerevisiae 288c	Our lab
S. cerevisiae BY4741	EUROSCARF
S. cerevisiae W303-1A	EUROSCARF
S. cerevisiae H1246α	(14)
**Plasmids**	
pCXSN	(9)
p426-GAL	(10)
pXDR	(9)
pYES2.1-LacZ	Invitrogen
p426-JcDGAT1	(10)
p426-JcDGAT2	(10)
pYES2.1-JcPDAT1	(11)
pWXY1.0	This study
pWXY3.0	This study
pWXY1.0-JcDGAT1	This study
pWXY1.0-JcPDAT1	This study
pWXY1.0-LacZ	This study
pWXY1.0-EGFP	This study
pWXY3.0-ScDGA1-EGFP	This study
pWXY3.0-ScDGA1	This study
pWXY1.0-JcDGAT2	This study
