# Supplementary material for: IRDL Cloning: A One-Tube, Zero-Background, Easy-to-Use, Directional Cloning Method Improves Throughput in Recombinant DNA Preparation
Source: PLoS One. 2014 Sep 22;9(9):e107907. doi: 10.1371/journal.pone.0107907 (PMC4171505; doi:10.1371/journal.pone.0107907)
Supplement: Table S2 — Primers in the study. (PDF) [file pone.0107907.s002.pdf]

Table S2. Primers in the study.

Name	Sequence (5' to 3')	Comments
TRP1-1	gtatgcttccttcagcactaccctAATTCGGTCGAAAAAAGAAAA	Forward primer to amplify TRP1 cassette
TRP1-2	ggacgctcgaaggctttaatttgcGGCAAGTGCACAAACAATACTTA	Reverse primer to amplify TRP1 cassette
COS1	GCAAATTAAAGCCTTCGAGCGTCCC	Forward primer to amplify vector p426-Gal
COS2	AGGGTAGTGCTGAAGGAAGCATACG	Reverse primer to amplify vector p426-Gal
ccdB-1	GCGCCTCGAGGATCCATGGCATGCTGTATGGGGCTTACTAAAAGCC	Forward primer to amplify ccdB cassette
ccdB-2	GCGCACTAGTGGTACCGAATTCAAGCTTGTATGGGTCGACCTGCAGACTGGC	Reverse primer to amplify ccdB cassette
URA3-1	tctagagaataggaacttcggaataAGTGCACCATACCACAGCTT	Forward primer to amplify URA3 cassette
URA3-2	gggacgctcgaaggctttaatttgcCCGCATAGGGTAATAACTGATA	Reverse primer to amplify URA3 cassette
COS3	TATTCCGAAGTTCCTATTCTCTAG	Reverse primer to amplify vector pWXY1.0, the forward primer use COS1
GFP1	GCGCGGTACCATGGTGAGCAAGGGC	Forward primer to amplify fragment *KpnI-EGFP-XhoI*, and also used as forward primer for colonies PCR
GFP2	GCGCCTCGAGTTACTTGTACAGCTCGTCCATG	Reverse primer to amplify fragment *KpnI-EGFP-XhoI*
GFP3	GCGCACTAGTATGGTGAGCAAGGGC	Forward primer to amplify fragment *SpeI-EGFP-NcoI*
GFP4	GCGCCCATGGTTACTTGTACAGCTCGTCCATG	Forward primer to amplify fragment *SpeI-EGFP-NcoI*
GFP5	GCGCGAATTCATGGTGAGCAAGGGC	Forward primer to amplify fragment *EcoRI-EGFP-BamHI*
GFP6	GCGCGGATCCTTACTTGTACAGCTCG	Forward primer to amplify fragment *EcoRI-EGFP-BamHI*
D1-1	GCGCGGTACCATGACGATTTTGGAGACCACT	Forward primer to amplify fragment *KpnI-JcDGAT1-XhoI*, and also used as forward primer for colonies PCR
D1-2	GCGCCTCGAGTCATCTTAATTCAGCATTGCC	Reverse primer to amplify fragment *KpnI-JcDGAT1-XhoI*
P1-1	GCGCGGTACCATGGCGATTTTGCGACGGA	Forward primer to amplify fragment *KpnI-JcPDAT1-XhoI*
P1-2	GCGCCTCGAGCTACAGCTGCAAGTTAATCCTTT	Reverse primer to amplify fragment *KpnI-JcPDAT1-XhoI*
P1-T	CACGGCTGGGTCAACTTTAGATCTTC	Forward primer for colonies PCR
L1-1	GCGCGGTACCATGATAGATCCCGTCG	Forward primer to amplify fragment

	TTTTACA	*KpnI-LacZ-XhoI*
L1-2	GCGCCTCGAGTCAATGGTGATGGTGATGATGA	Reverse primer to amplify fragment *KpnI-LacZ-XhoI*
L1-T	TGCGCGATCAGTTCACCCGT	Forward primer for colonies PCR
DGA1-1	GCGCACTAGTATGTCAGGAACATTCAATGATATAA	Forward primer to amplify fragment *SpeI-ScDGA1-AscI*
DGA1-2	GCGCGGCGCGCCCCAACTATCTTCAATTCTGC	Reverse primer to amplify fragment *SpeI-ScDGA1-AscI*
GFP-7	GCGCGGCGCGCCTATGGTGAGCAAGGGCG	Forward primer to amplify fragment *AscI-EGFP-XhoI, the reverse primer use GFP-2*
P1	CATGGTAGGCGGAGATGGC	Forward primer to amplify fragment *JcDGAT2*
P2	TCGAGTCAAAGGATTTCAAGTTTAAGGTC	Reverse primer to amplify fragment *JcDGAT2*
P3	AATTCATGGTAGGCGGAGATGGC	Reverse primer to amplify fragment *JcDGAT2*
P4	GTCAAAGGATTTCAAGTTTAAGGTC	Forward primer to amplify fragment *JcDGAT2*
CYC1	GCGTGAATGTAAGCGTGAC	Sequencing primer of pWXY1.0 or pWXY3.0 and Reverse primer of colonies PCR.
GAL1	AATATACCTCTATACTTTAACGTC	Reverse sequencing primer of pWXY1.0 or pWXY3.0

The underlined bases encode restriction enzyme site. The lowercase letter showed overlapping sequence used in yeast recombination.
