# Supplementary material for: IRDL Cloning: A One-Tube, Zero-Background, Easy-to-Use, Directional Cloning Method Improves Throughput in Recombinant DNA Preparation
Source: PLoS One. 2014 Sep 22;9(9):e107907. doi: 10.1371/journal.pone.0107907 (PMC4171505; doi:10.1371/journal.pone.0107907)
Supplement: Materials and Methods S1 — (PDF) [file pone.0107907.s003.pdf]

Supplemental Materials and Methods S1

pWXY1.0

A 4252-bp DNA fragment containing 2 μ origin, Ampicillin resistance cassette, pBR322 origin, Gal1 promoter, MCS and CYC1 terminator was amplified using plasmid p426-Gal as template with primers COS1 and COS2. An 871-bp DNA fragment containing a *TRP1* cassette was amplified using *S. cerevisiae 288c* genomic DNA as template with primers TRP1-1 and TRP1-2. The primers TRP1-1 and TRP1-2 provide the *TRP1* cassette with 24-bp ends that are homologous to the amplified vector. The two fragments were purified from agarose gels and co-transformed the *S. cerevisiae* W303-1A. The transformants were selected using synthetic complete plates lacking tryptophan incubated at 30 °C. The total DNA (including the rescued plasmids) was extracted from all colonies that were scraped from the plates, and then was transfomed into *E. coli* Trans1-T1, and plated on LB plates containing 100 μg/mL ampicillin (Amp^+). The resulting plasmid was confirmed by restriction digestion and designated pX3a. A 732-bp DNA fragment containing a cassette of *XhoI-BamHI-NcoI-SphI-ccdB-HindIII-EcoRI-KpnI-SpeI* was amplified using plasmid pCXSN as template with primers ccdB-1 and ccdB-2, and was digested with SpeI and XhoI, and subsequently inserted in the respective sites of the pX3a vector. The resulting plasmid was designated pWXY1.0.

pWXY3.0

An 1173-bp DNA fragment containing a *URA3* cassette was amplified using plasmid p426-Gal as template with primers URA3-1 and URA3-2. A 4610-bp DNA fragment minus *TRP1* cassette was amplified using plasmid pWXY1.0 as template. The two fragments were recombined in *S. cerevisiae* BY4741 using the same procedure as above and the resulting plasmid was designated pWXY3.0.

pWXY1.0-EGFP, pWXY1.0-JcDGAT1, pWXY1.0-JcPDAT1, pWXY1.0-LacZ

To generate the construct pWXY1.0-EGFP, the coding region of the *EGFP* gene from the plasmid pXDG was PCR-amplified (35 cycles at 95 °C for 20 s, 55 °C for 20 s, and 72 °C for 30 s with TransStart^{TM} FastPfu) using the primers GFP1 and GFP2 (for KpnI and XhoI digestion),

GFP3 and GFP4 (for SpeI and NcoI digestion), GFP5 and GFP6 (for EcoRI and BamHI digestion). The amplified *EGFP* genes were purified by electrophoresis and inserted in the plasmid pWXY1.0 by standard IRDL cloning procedure using the respective restriction enzymes. The integration of the *EGFP* gene into the vector was confirmed by PCR analysis using the primers GFP1 and GFP2 or by DNA sequencing analysis using the primers CYC1 and GAL1 (Table S2).

To generate the construct pWXY1.0-JcDGAT1, pWXY1.0-JcPDAT1 and pWXY1.0-LacZ, the coding region of *JcDGAT1*, *JcPDAT1* and *LacZ* genes were amplified form p426-JcDGAT1, pYES2.1-JcPDAT1, and pYES2.1-LacZ with respective primers D1-1 and D1-2, P1-1 and P1-2, L1-1 and L1-2. The *JcDGAT1*, *JcPDAT1* and *LacZ* genes were inserted in the plasmid pWXY1.0 by standard IRDL cloning procedure using KpnI and XhoI digestion. PCR verification and sequencing as described above.
